# 

**Published:** 1812-02

**Authors:** 


### MONTHLY CATALOGUE OF MEDICAL BOOKS.

BARCLAY's (Dr. John) Description of the Arteries, with copious Notes. 18mo.—Bryce and Co.

Cullen's (William, M.D.) Nosology; or, Diseases arranged in their Classes, Orders, Genera, and Species, with accurate Definitions. 18mo.—Longman and Co.

### *NOTICES TO CORRESPONDENTS.*

*We acknowledge to have received from Dr. Hossack, of New York, "Observations on Croup," with an "inaugural" Dissertation on Mercury, by John W. Francis, A.B.*

*In the present difficulty we find in procuring periodical and other Publications from America, we feel additional obligations to our Friends who honour us with Communications from that Country.*

*We are indebted to a learned and ingenious Physician for the first part of the Collectanea Medica, in our present number; and hope in future, from our own resources, and those of our friends, to continue an article which may prove interesting to the Public.—Mr. Shute, of Bristol, informed us of his Lectures too late to announce them in the usual way.*

*We have been favored with communications from A. Copland Hutchinson, M.D.—J. Ramsbotham, M.D.—Dr. B.—From Mr. J. Scott—Dr. James Bradley—L. N.—An Essex Practitioner, &c. &c.*

*It gives us pleasure to state, in contradiction to the reports in circulation, that Letters have been received from Dr. Willan, dated the 20th and 21st of December, in which he describes a gradual amelioration of his health, since his arrival at Madeira, in the beginning of that month, after a passage of only ten days from Portsmouth.*

*We request our numerous Correspondents, in future, will address their Favors to the Publisher, Sir R. PHILLIPS, No. 47, Ludgate Hill; or to J. ADLARD, No. 23, Bartholomew Close.*

